# Drift detection on feature attributions for monitoring visual reinforcement learning models in maritime port surveillance

**DOI:** 10.12688/openreseurope.22116.1

**Published:** 2026-01-02

**Authors:** Francisco Javier Iriarte, Beatrice Azoubel, Adrián Carrizo-Pérez, Andrés Chica Linares, Luis Unzueta, Ignacio Arganda-Carreras

**Affiliations:** 1 Fundación Vicomtech, Basque Research and Technology Alliance (BRTA), Donostia/San Sebastián, Gipuzkoa, 20009, Spain; 2 Department of Computer Science and Artificial Intelligence, University of the Basque Country (UPV/EHU), Bilbao, Basque Country, 20018, Spain; 3 INDRA Sistemas S.A., Alcobendas, 28108, Spain; 4 Donostia International Physics Center, San Sebastián, Basque Country, 20018, Spain; 5 Biofisika Institute (CSIC-UPV/EHU), Leioa, Bizkaia, 48940, Spain; 6 Ikerbasque, Bilbao, Basque Country, 48009, Spain

**Keywords:** Visual Reinforcement Learning, Drift Detection, Explainable AI, Continuous Model Monitoring, Maritime Port Surveillance

## Abstract

**Background:**

Maritime activity is expanding globally, increasing the demand for robust port security systems capable of detecting illegal trafficking. Due to the growing sophistication of smuggling methods, law enforcement agencies require advanced surveillance and prevention technologies such as those developed in the SMAUG project. In this context, initiatives such as the SMAUG project aim to deliver integrated surveillance capabilities coordinated by a high-level deep reinforcement learning (DRL) decision-making system that operates on image-based environmental representations. Despite their effectiveness, DRL models are closed-boxes, complicating continuous model monitoring (CMM). Conventional drift detection captures shifts in input or output distributions yet often fails to explain underlying problems. Explainable AI (XAI) techniques can provide a complementary approach with insights into the agent’s inner workings, enabling monitoring of the concept rather than just the data.

**Methods:**

We propose FADMON, an XAI-driven concept drift detection method for image-based models. FADMON performs statistical drift tests on feature attributions to detect deviations in learned policies. We demonstrate how FADMON can enhance CMM with a three-stage model monitoring architecture that enables semi-supervised explainable model monitoring. We validate our approach with SMAUG’s decision-making DRL model on a simulated maritime port surveillance environment under multiple unforeseen scenarios.

**Results:**

FADMON consistently flags drift on all drifted scenarios with mean p-values of 0.000 with no variance trough 30 repetitions, with lower mean p-values (0.553±0.215) on non-drifted scenarios with respect to other established drift detection methodologies such as prior probability shift detection (0.65 ± 0.000), though well above the standard 0.05 threshold.

**Conclusions:**

FADMON can add an explainability layer to the monitoring system while also supporting detection of changes in the underlying interpretation of the input data by the model, monitoring the concept rather than the data, while matching established drift detection methods metrics-wise.

## Plain Language Summary

This article shows how it is possible to monitor an AI model interpretation of the data it is being fed, rather than monitoring only the data itself, to improve our capability to understand how an AI model is behaving while it works. This allows us to detect changes in the way it is behaving, as well as understand these changes better, in order to detect more quickly when the model is not behaving correctly and fix it. In this article, we apply this approach to a maritime port surveillance environment, testing it under different simulated scenarios, such as overabundance of obstacles in the port or malicious attacks on the vessels, to see if the method meets its potential. We conclude that it does, with certain caveats such as the need of stronger hardware, resulting in a good balance between detection of irregular AI model behavior and interpretability of said behavior.

## Introduction

Global maritime activity continues to expand in both scale and complexity, with extended trade routes and increased port throughput across major global hubs ^1^. This sustained expansion, however, has been accompanied by a surge in maritime trafficking and smuggling operations. Recent studies by Europol and the European Monitoring Centre for Drugs and Drug Addiction ^2^ highlight a marked increase in the use of maritime routes, particularly containerized cargo and coastal vessels, for the trafficking of drugs and other illicit goods into Europe.

In response to these growing challenges, the European Union has supported several research and innovation initiatives aimed at enhancing maritime domain awareness and port security. The COMPASS2020 project demonstrated the operational integration of manned and unmanned platforms to extend surveillance coverage and reduce reaction times in coastal environments ^3^. Similarly, the RAPID project advanced the concept of risk-aware, autonomous inspection by combining unmanned surface and aerial vehicles for real-time hull and infrastructure assessment ^4^. Ongoing projects like UNDERSEC further contribute to this line of research by developing a modular underwater security architecture leveraging robotic assets for persistent port protection ^5^. Building upon these efforts, the SMAUG project introduces a comprehensive framework that integrates acoustic sensing, rapid sonar hull scanning, and high-resolution underwater inspection to detect and characterize concealed threats such as submersible vessels or smuggling devices ^6^.

SMAUG’s integrated architecture includes a high-level decision-making module that manages the system in real time. Deep reinforcement learning (DRL) has facilitated the design of these systems, in which RL agents learn to make optimal decisions through interaction with complex, partially observable environments. In particular, visual RL, where policies are trained end-to-end from pixel inputs, is being increasingly adopted in safety-critical and high-stakes domains, including robotics, autonomous navigation, and maritime port surveillance ^7^. However, DRL models, like any other deep neural network (DNN) model, are often opaque and treated as closed boxes ^8^. This opacity hinders the ability of developers and stakeholders to diagnose errors, increasing the risks and consequences of policy failures in high-risk applications.

These consequences are further amplified because DNNs are prone to performance degradation after deployment. This is commonly caused by shifts in the environment compared to the conditions seen during training, a phenomenon referred to as data drift. Moreover, the relationship between observations and the RL agent’s chosen actions may also evolve over time, either because of data drift or due to the emergence of new behavioral patterns in the environment. This is typically described as concept drift ^9^. Both forms of drift undermine the reliability of RL agents, particularly in dynamic real-world scenarios such as maritime ports.

The MLOps paradigm introduces Continuous Model Monitoring (CMM) as a mechanism to track deployed models and identify failure cases in order to update policies and sustain performance ^10^. CMM is an important procedure that helps ensuring robust and trustworthy AI systems. For image-based models such as visual RL, however, this process is even more challenging than for regular supervised models, since reward signals are sparse, action distributions are high-dimensional, and access to ground-truth performance is limited ^11^.

Classical drift detection methods such as the Kolmogorov–Smirnov (KS) test ^12^ or Maximum Mean Discrepancy (MMD) ^13^ enable unsupervised monitoring by analyzing shifts in the input or output distributions. Yet, these techniques only consider environment states or policy outputs in isolation and do monitor nor provide visibility into the RL agent’s internal decision-making process, making concept drift detection more challenging. Furthermore, fields like maritime port surveillance fall into the law enforcement and critical infrastructure categories under the European Commission’s AI Act ^14^ and thus could be considered as high-risk AI systems if they meet certain conditions such as it profiling individuals or replacing human assessment. Therefore, any AI system used in these scenarios should comply with robustness and transparency standards that are not feasibly achieved with drift detection methods alone.

Explainable AI (XAI) methods such as SHAP ^15^ or Integrated Gradients ^16^ can provide feature attribution-based explanations that make policy decisions more transparent. By exposing the specific parts of an observation that drove an RL agent's action, XAI offers the necessary visibility into the decision boundary that is entirely missing from classical drift detection methods (like KS or MMD). As such, combining classical statistical drift detection with feature attributions holds significant potential to detect concept drift and improve compliance in high-risk applications. Recent articles have examined this combination for tabular models ^17– 19^, but this approach is yet to be adapted to image-based models.

In this paper we propose FADMON (Feature Attribution Drift MONitoring), a semi-supervised, explainability-driven approach for concept drift detection of visual RL agents. By combining statistical drift detection with feature attribution-based explanations, FADMON supports unsupervised detection of concept drift while also enabling health reports and diagnostics of the model’s decision-making process. We validate our approach directly under SMAUG’s decision-making environment and RL agent, testing out methodology on a representative component of a maritime port surveillance system and verifying improvements on the robustness, transparency and maintainability of the system. Our contributions are as follows:

- FADMON, a semi-supervised concept drift detection method for visual RL that integrates statistical tests with explainability techniques.
- An XAI-based semi-supervised model monitoring architecture that integrates FADMON to raise automatic alerts of data, concept and probability drift while offering online visualization of policy explanations.
- An empirical validation of the method through experiments on a simulated visual RL-based maritime surveillance environment under diverse scenarios.

## Related work

DRL combines reinforcement learning with DNNs to learn policies and value functions directly from high-dimensional inputs, enabling end-to-end decision-making systems from images or raw sensors. Early breakthroughs like Deep Q-Network (DQN) used convolutional networks with experience replay and target networks for discrete control, establishing practical stability tricks that informed later algorithms ^20^. This was followed by policy-gradient and actor–critic families for continuous action spaces, such as Asynchronous Advantage Actor-Critic (A3C), which leverages parallel actors and advantage estimation for more stable updates ^21^ and Proximal Policy Optimization (PPO), which refines on-policy learning with a clipped surrogate objective to prevent destructive step sizes while retaining sample efficiency ^22^. In visual domains, representation learning with Convolutional Neural networks (CNN) and, increasingly, Vision Transformers, together with auxiliary or self-supervised objectives, improves feature quality, stabilizes training, and reduces data requirements. Temporal encodings, frame stacking, and lightweight recurrence are often combined to exploit dynamics in video-based inputs ^7^.

Deep reinforcement learning is increasingly deployed in high-risk, safety-critical domains. Regarding maritime surveillance and law enforcement, DRL has been explored for port and open-sea coverage to detect abnormal vessel behaviors, support wide-area search, track targets, and better allocate resources under challenging sensing conditions ^23^. Beyond the maritime domain, DRL coordinates multi-Unmanned Aerial Vehicle (UAV) surveillance by learning cooperative policies that improve reliability and coverage despite communication and dynamics uncertainties ^24^ as well as forensic investigation, where it has been proposed to streamline workflows by prioritizing evidence and adapting search strategies in complex scenes ^25^. In autonomous driving, recent surveys document DRL’s role in the control and decision-making systems at the core of self-driving vehicles ^26^.

Production-level use of DRL (as well as AI systems in general) has drawn attention to certain drawbacks that DNNs present after being deployed, which directly threaten their trustworthiness and viability: DNNs tend to lose accuracy over time ^27^, and are not understandable by humans due to their complexity and "black-box" behavior ^28^.

The phenomenon of models performing worse over time is often referred to as model drift, which is often caused by two key factors: data drift (or covariance shift), which refers to the distribution of the observed data changing over time, and concept drift, referring to changes in the relationship between input and output data of the model ^29^. Drift detection is the process that aims to detect model drift when it occurs. It has many implementations, such as statistics-based tests like the Kolmogorov-Smirnov test ^12^, Cramér-von Mises ^30^, or Maximum Mean Discrepancy (MMD) ^13^, or model-based techniques such as ADWIN or drift detection method (DDM) ^31^. Drift detection methods can be supervised, unsupervised or semi-supervised depending on whether labelled data (and thus manual annotation) is required, not required or partially required respectively ^32^.

Drift detection is a key component of CMM. Unsupervised drift detection is key in production environments; as it is impractical to label great amounts of operational data continuously and in short time. Still, while useful, drift detection methods do not provide insight into how a model responds to changes in data, making them unable to address the difficulty of comprehending why models make mistakes. Performance metrics such as accuracy, precision, recall, and F1-score offer some level of understanding, but as models increase in size and complexity, these metrics alone may be inadequate, particularly for DNNs ^33^.

To better understand the reasoning behind a DNN's decisions and diagnose the factors that lead to mistakes, XAI methods aim to provide additional information from the model via explanations. These explanations can be either intrinsic from inherently interpretable models ^34, 35^ or be provided from external methods that generate them in a post-hoc manner ^36^. They can be also classified as model-specific ^34, 35^ or model-agnostic ^31, 37^ if the method is applicable to a specific architecture or to any architecture, respectively. Finally, XAI methods can vary in the scope of their explanations: global methods aim to explain the model's general behavior ^34, 35^, while local methods generate explanations for specific predictions, giving insight on why the model reached that answer ^36, 37^. XAI methods have been applied successfully to many Computer Vision architectures, e.g., CNNs ^38^ and Vision Transformers (ViT) ^39^.

XAI methods can convey explanations in multiple formats, such as feature attributions, which assign each input feature an importance score for how much it influenced a given output, or counterfactual explanations, which generate slightly modified inputs that radically change the original output ^40^. Extending feature attributions, attribution (or saliency) maps visualize how input features contribute to an image-based model’s prediction, typically as pixel-level heatmaps. Gradient-based methods include Integrated Gradients ^16^, which estimate contributions by accumulating integrals through the network. G-DeepSHAP ^41^ propagates Shapley-value attributions through a composition of differentiable layers/components, providing efficient attributions for neural networks and model stacks. Some methods like Grad-CAM ^36^ and its follow-ups (e.g., Grad-CAM++ ^42^, Eigen-CAM ^43^) specialize in CNN architectures to produce attribution maps for specific output classes. For model-agnostic analysis, KernelSHAP ^15^ can compute attribution maps by computing the model’s output over a set of modified inputs that omit specific features, aggregating output changes with Shapley’s game theory. B-cos makes attributions interpretable by design by replacing standard linear layers with a B-cos transform that enforces input–weight alignment, so a model’s forward pass yields faithful, human-readable saliency that coherently sums contributions across layers ^34^.

However, feature attribution methods have limitations independently on the method used to approximate them. SHAP values - same as its inspiration, the Shapley values - can be misinterpreted, and it's even possible to create intentionally misleading interpretations, reducing trustworthiness of explanations that are implemented to improve it in the first place. These methods are also known to make feature independence assumptions, as well as only highlight correlations that do not imply causality. Finally, deterministic implementations like KernelSHAP are slow and unsuitable for real-time monitoring. Global explanations are particularly affected, since generating them would require computing several local values ^44^.

Feature attributions could be used to monitor the model's interpretation of the input data, complementing monitoring of the data itself. Recent studies have successfully implemented drift detection on SHAP values to monitor ML models for tabular data ^17– 19^ but, to the best of our knowledge, its applications for image-based models such as visual RL have not yet been explored.

## Methodology

**Figure 1.**
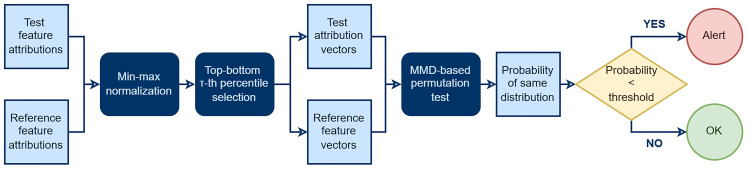
Flow diagram of FADMON, illustrating how it detects concept drift by performing drift detection on feature attributions.

Figure 1 shows the flow diagram of FADMON, our proposed attribution-based concept drift detector. The method takes pixel-level feature attributions of a reference dataset and a batch of test data, previously computed using methods such as SHAP or Integrated Gradients. Then, min–max normalization is applied on all attributions using only the reference set’s extremes. This sets all values to a fixed scale based on the reference attributions, providing a stable baseline and amplifying deviations in the test attributions. Next, to reduce the influence of the pixels with near-zero activations which dilute distributional differences, we perform top-bottom percentile selection. We retain only the upper and lower τ-th percentiles of this feature attributions, where τ ∈ (0,1) is the percentile threshold. These values are sorted to form fixed-length feature vectors, discarding spatial information since it is not relevant for feature attributions. Finally, we compute the Maximum Mean Discrepancy (MMD) between the reference and test vectors, yielding a statistically meaningful metric of the similarity of the distributions from which reference and test data came from. This value is compared against a predefined threshold to create concept drift alerts: If the similarity of the original distributions is lower than the threshold, FADMON flags concept drift on the current test data batch.

MMD is an integral probability metric that measures the distance between the distributions of two samples by mapping them with a kernel into a reproducing kernel Hilbert space (RKHS) and computing the norm of the difference between their mean embeddings ^13^. In practice, it evaluates pairwise kernel similarities within and across the two samples; with a characteristic kernel (e.g., Gaussian RBF), MMD equals zero if and only if the underlying distributions are identical. MMD is multivariate and non-parametric, making it well suited to high-dimensional signals such as images or attribution vectors, and is widely used both as a loss/cost for training machine learning models (e.g., GANs) ^45^ and for drift detection. Because the method is kernel-based, one can tailor sensitivity to different forms of shift by choosing the kernel and its hyperparameters (e.g., bandwidth in the Gaussian RBF kernel). These properties make MMD a good fit for FADMON, though any statistical metric that measures distributional differences and supports high-dimensional feature vectors can replace or complement MMD.

**Figure 2.**
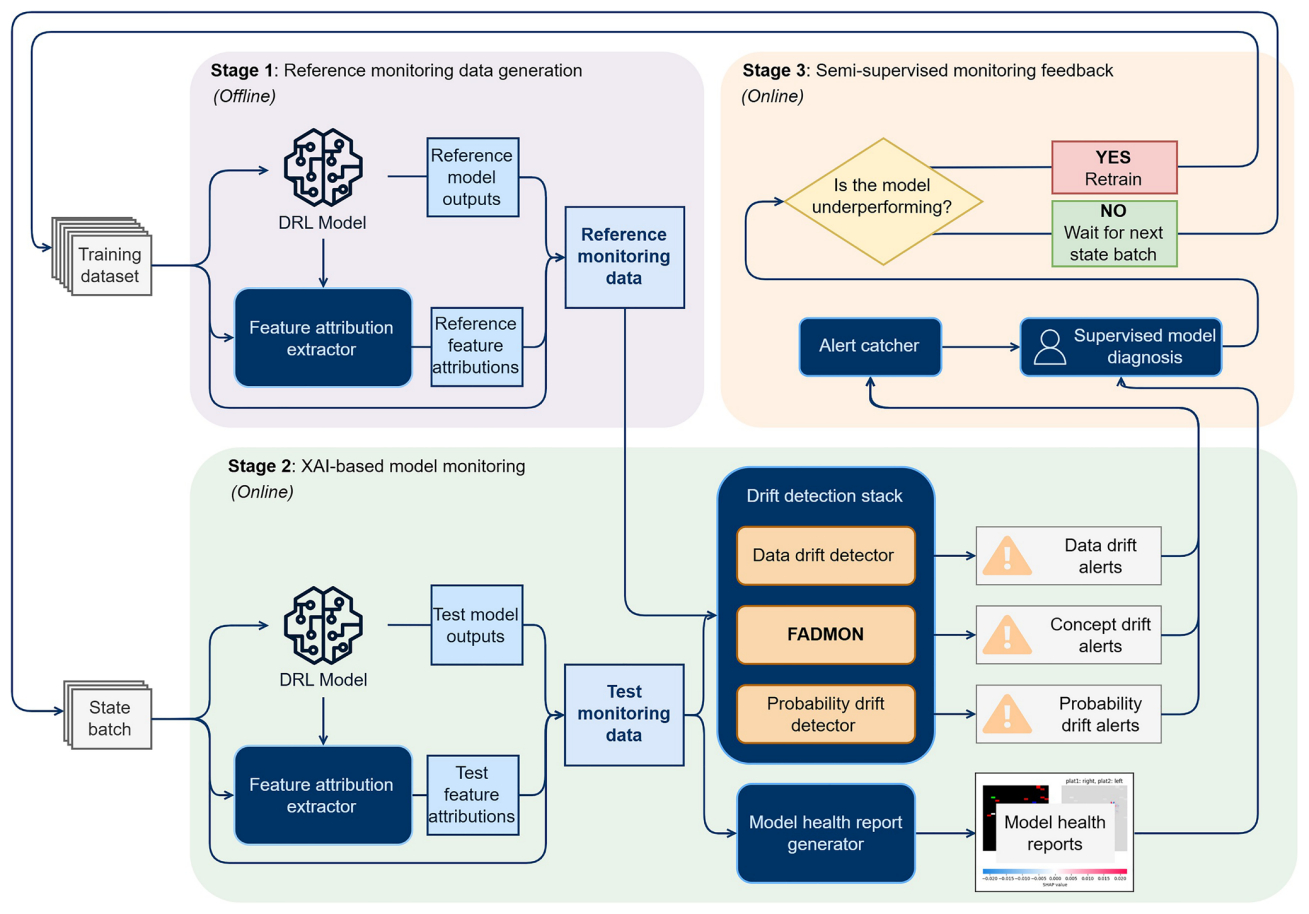
XAI-based semi-supervised model monitoring architecture. Stage 1 is done offline and once per model version, generating reference monitoring data. Stage 2, the main stage, performs online monitoring that creates drift alerts and user-friendly model health reports. Stage 3 involves a user that analyzes monitoring data in a semi-supervised way and decides if model retraining is necessary.

To better specify how FADMON could enhance CMM, we will explain next how to integrate it in a model monitoring architecture. Figure 2 illustrates this three-stage architecture that enables XAI-based monitoring of a deployed DRL agent. Stage 1 builds reference monitoring data, which contains the values corresponding to the expected behavior of the model. Stage 2 takes incoming state batches and performs online model drift detection including concept drift detection using FADMON. The feature vectors computed for FADMON are also leveraged to create user-friendly model health reports. Stage 3 closes the loop with semi-supervised feedback from an expert user, potentially scheduling a model retraining.

The workflow is kickstarted by a new model version. The process begins with the reference monitoring data generation stage, where a working copy of the model as well as a feature attribution extractor process the training data on which the model was originally trained. This generates a pack of data consisting of three types of monitoring signals: input states (images), model outputs (probability vectors), and pixel-level feature attributions. Though any feature extraction method can potentially work, a low latency method like Integrated Gradients or an interpretable-by-design model such as B-cos are recommended, since the same method is also deployed in near-real-time at the next stage. Stage 1 is done offline, either in parallel or before the model is deployed, and is only activated once per model version since the reference monitoring data needs to be generated only the first time.

The second stage, XAI-based model monitoring, is the main stage of the architecture and the one integrating FADMON. This stage periodically takes batches of current states the DRL model is exposed to, processing them with a working copy of the model and the feature extraction method, thus obtaining test monitoring data with the same structure as the reference. This data is passed through the drift detection stack, containing three drift detectors that detect distributional changes: The data drift detector, which analyzes input images, the probability drift detector, which analyzes probability vectors, and FADMON, which analyzes feature attributions. This stack generates soft-real-time alerts, enabling swift detection of data, probability, and concept drift. In parallel, the test monitoring data is reused to create model health reports: User-friendly visualizations of every piece of relevant information for model monitoring. These reports include the original input, output, and attribution maps, as well as any additional information relevant to the real-time monitoring of the model, such as date, time and/or operation status. Since local feature attributions are used, a report can be generated for each observed state, as well as aggregated reports for global explanations.

Finally, the semi-supervised monitoring feedback stage introduces a semi-supervised feedback loop in which human experts can react to alerts and diagnose model performance by examining statistical metrics and the model health reports, being able to confidently determine whether readjustment actions are required. All alerts produced in Stage 2 are routed to an alert catcher module that filters, aggregates, and prioritizes them. When a relevant incident is detected, the alert catcher triggers a supervised model diagnosis step. If the diagnosis concludes that the DRL policy is underperforming or that the detected drift poses a considerable risk, model retraining is kickstarted, involving collection of the data that triggered the drift alert in the first place and returning to Step 1 after model and training data are updated. If the analysis concludes that the model is still able to function correctly, the architecture returns to the start of Step 2, waiting for a new state batch to arrive to start the monitoring process again.

## Experimental results

### Visual RL model

For our experiments, we make use of the Proximal Policy Optimization (PPO) model architecture. PPO is an on-policy, actor–critic reinforcement learning algorithm that updates the policy by maximizing a clipped surrogate objective, which constrains each gradient step to stay close to the previous policy. This stabilizes training while still allowing relatively large policy updates and has become a standard baseline for continuous and discrete control tasks ^22^.

In SMAUG, PPO is deployed as a high-level decision-making module that coordinates a fleet of surveillance vessels within a port. The policy recommends routes for each vessel that aim to minimize traversal time and fuel consumption while avoiding static obstacles and other moving vessels. The system operates in a target analysis mode, in which vessels are required to move towards specific locations to perform detailed inspection of regions of interest in the port.

SMAUG’s decision-making system operates on pixel level representations of the port’s state. Following this specification, we train and evaluate an instance of an image-based PPO architecture that leverages a convolutional backbone to extract features from pixel-level representations, outputting a probability vector of discrete motion commands (up, down, left, right), indicating the direction in which the vessel should move at the next decision step.

### Dataset

**Figure 3.**
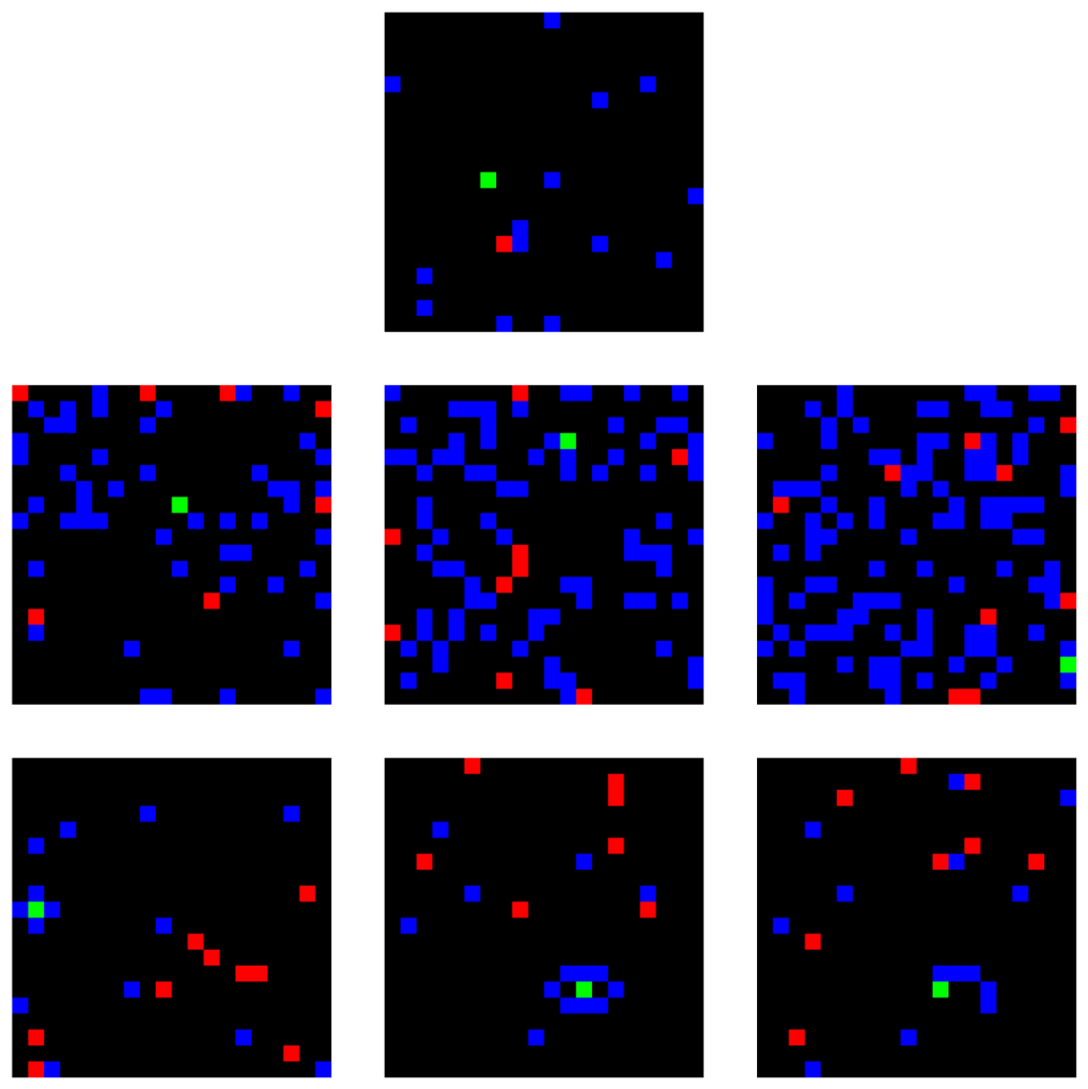
Examples of the pixel-level representations that serve as model input. Reference (top), obstacle density increase (middle), and vessel confinement (bottom). Blue pixels represent obstacles, red pixels represent targets, green pixels represent vessels, and black pixels represent traversable areas.

We generate a series of synthetic datasets of the model’s expected pixel-level states in multiple scenarios, both under normal conditions and under unexpected situations. We create these datasets by simulating vessel positions, obstacles, and regions of interest and render them into image-like states that match the input format of the PPO agents. In these states, blue pixels represent obstacles that are not traversable and should be avoided, red pixels represent targets the vessels need to reach, green pixels represent vessels, and black pixels represent traversable areas (in this case, obstacle-free water). To introduce realistic model drift in maritime port environments, we create two unexpected or drifted scenarios. These drifted configurations are used to generate states that depart from the reference distribution, emulating operational changes in the environment that the model has not seen during training. All scenarios are simulated during a total of 300 timesteps, resulting in 300 pixel-based representations, for a total of 2100 samples. Figure 3 illustrates examples of both reference and drifted states for the target analysis model.

The first drift scenario is obstacle density increase, where the port environment is filled with more obstacles than the model has seen during training. This scenario immediately introduces realistic data drift, as the drifted input representations differ from the ones used for model training. This data drift causes, in turn, changes in the optimal policy, since older optimal routes may be riskier, more costly, or even impossible if objective are unreachable, inducing concept drift. We create three datasets of this drifted scenario with variable increases in obstacle density. Three variants of this scenario have been creating, each with increasing obstacle density.

The second drift scenario, vessel confinement, involves partially or completely surrounding vessels with obstacles and effectively locking them in place or greatly restricting its movement options, simulating an accident with other elements of the port or an intentional attack. While this scenario also introduces data drift in the increase of targets over time, this represents a sharper alteration in the expected reward of the decisions taken by the model: actions that were previously safe become impossible to make or even dangerous, leading to a stronger concept drift in the optimal behavior. Three variants have been created as well: One where the vessel is fully surrounded with no possible movement; a second where it is fully surrounded but retains a narrow area to move; and a third where it is not fully surrounded, but obstacles are arranged so that the policy cannot reliably guide the vessel out of the blocked region.

### Feature attribution extraction method

We make use of Expected Gradients, commonly known as GradientSHAP, to approximate SHAP values for DRL models. SHAP values quantify how much each feature contributes (positively or negatively) to a model’s prediction, thus serving as feature attributions. The method makes use of a background set of input data, which constitutes the method’s baseline samples. These samples are then used to generate interpolated inputs and average the output’s gradients with respect to theirs, resulting in approximate SHAP values. GradientSHAP is notably fast, making it especially suited for image-based deep differentiable models, such as the ones use in Visual RL that comparatively have a greater number of input features than tabular models. This method is also known to compute smooth attributions that are consistent between executions, if a correct background is provided.

The size of the background used by GradientSHAP is unique for each case and needs to be calibrated. This background must be sufficiently large to maintain the original distribution of the training dataset, while not being excessively large, as this can significantly increase the computational cost of the explanation process. We estimate a suitable background size as follows: For a given model and candidate background size, we repeatedly (100 times) build a background by randomly sampling training instances. We then compute feature attributions using GradientSHAP, yielding a set of attributions that we normalize and use to compute a variance matrix over features and pixel positions. We then average this matrix to obtain a single mean variance score for that background size. Comparing these scores across sizes allows us to assess how background size affects the stability of the feature attributions and find the value that yields the highest precision over computational cost. Based on this analysis, a background size of 200 was selected for the experiments.

### Experimental workflow

To test FADMON under a relevant scenario and compare it to other established drift detection procedures, we compare the obtained p-values and MMD distances against a data drift and a probability drift method. All drift detectors are tested on the three variants of the drifted scenarios described in the Dataset section, as well as on an undrifted dataset with similar distribution to the training dataset to evaluate the method’s robustness.

We configure FADMON with a percentile threshold τ of 0.025 to receive feature attributions from a GradientSHAP instance with a background size of 200. To create our data drift detection workflow, we follow Rabanser *et. al.*’s proposed methodology, consisting of an untrained autoencoder to reduce input dimensionality followed by a statistical test on the resulting feature vectors ^46^. Probability drift is detected by directly performing the statistical test on the probability vectors that the model outputs. To compare the three approaches fairly, we employ MMD with an RBF kernel as the statistical test for all three drift detectors. Equity is also ensured by running the experiments for all detectors under multiple kernel bandwidth values, ranging from 0.01 to 1000, and selecting the value that maximizes the p-value difference of undrifted vs drifted datasets. Due to the randomness introduced by UAEs for data drift detection and by feature attribution approximations of GradientSHAP, each drift detection experiment is further repeated 30 times to obtain the mean and variance of each detector’s p-values.

## Results

The results of the experiment are illustrated in Table 1.

**Table 1.** Mean and variance of the p-values for each drift detection method, obtained by performing each experiment 30 times.

	Undrifted dataset	Obstacle density drifted scenario 1	Obstacle density drifted scenario 2	Obstacle density drifted scenario 3	Vessel confinement scenario 1	Vessel confinement scenario 2	Vessel confinement scenario 3
Data drift detection	0.757±0.217	0.059±0.086	0.007±0.02	0.003±0.006	0.132±0.93	0.066±0.093	0.209±0.135
Probability drift detection	**0.65±0.000**	0.008±0.000	**0.000±0.000**	**0.000±0.000**	**0.000±0.000**	**0.000±0.000**	**0.000±0.000**
**FADMON (ours)**	0.553±0.215	**0.000±0.000**	**0.000±0.000**	**0.000±0.000**	**0.000±0.000**	**0.000±0.000**	**0.000±0.000**

All methods correctly identify the undrifted dataset as originating from the same distribution as the reference, yielding p-values well above the standard 0.05 significance threshold. Among them, FADMON exhibits the lowest mean p-value on undrifted data which, although still safely non-significant, suggesting a certain oversensitivity to minor distributional changes. Still, FADMON consistently detects all drift scenarios with zero variance across runs, with all reported p-values numerically equal to 0 with no variance. This behavior represents a clear improvement over the data drift detector, which fails to flag drift in scenarios where changes in the input distribution are more subtle, such as the vessel confinement scenarios. These observations indicate that FADMON is more focused on the underlying concept drift, relying less on direct input distribution comparison and more on changes in the model’s attribution patterns.

**Figure 4.**
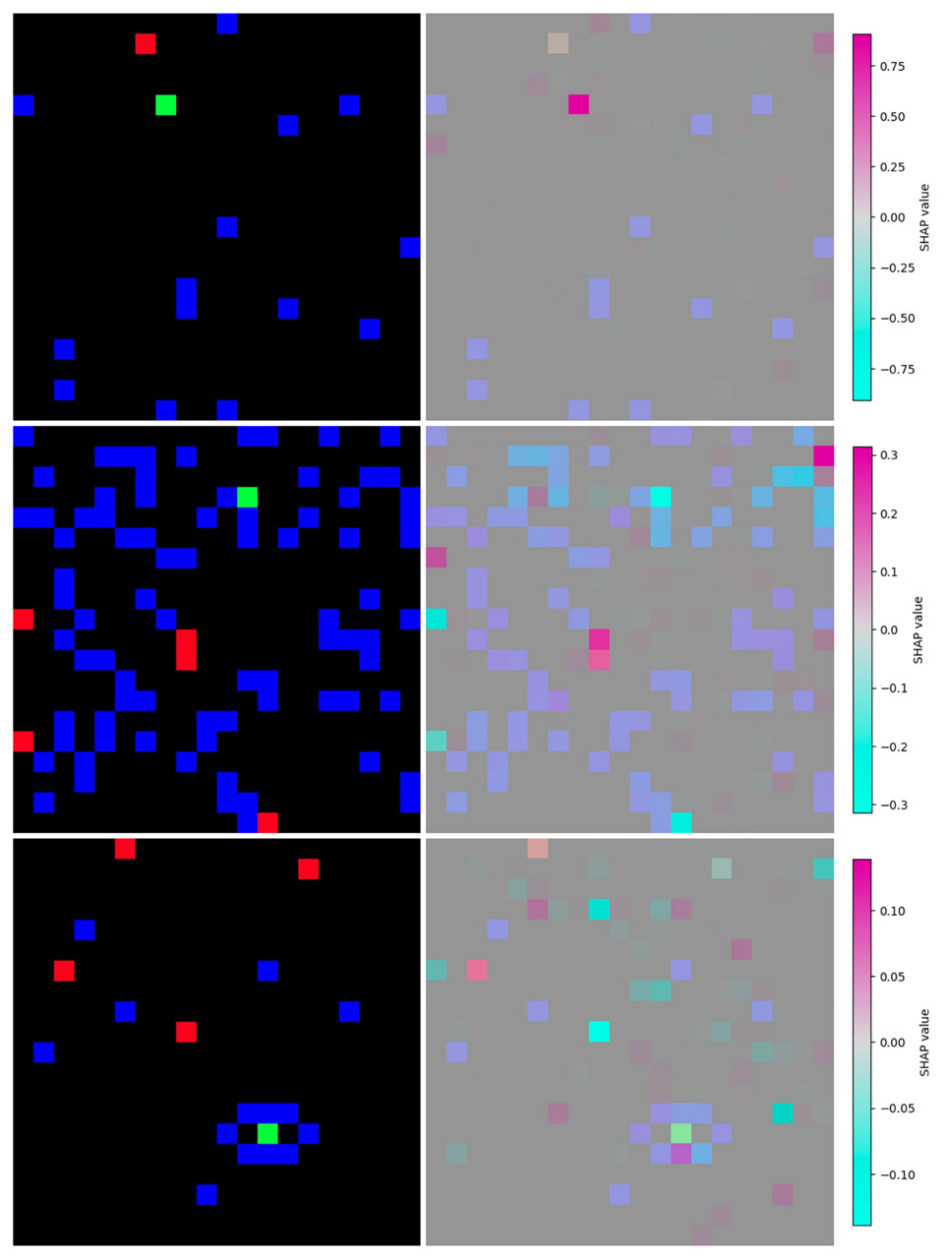
Examples of image-based model inputs (left) and visualizations of feature attributions computed by GradientSHAP (right) for the undrifted scenario (up), obstacle density increase scenario (middle) and vessel confinement scenario (bottom). Feature attributions show how the models interprets the data differently under the drifted scenarios, showing less overall feature activation strength as well as mixed positive-negative activations on targets.

The probability drift detector matches FADMON’s detection performance on the drifted scenarios and yields slightly higher mean p-values, with zero variance, on the undrifted dataset. This suggests that probability drift detection remains a strong baseline in terms of pure drift detection performance when only output distributions are considered. However, probability vectors are not inherently interpretable and therefore provide limited support for comprehensive supervised model monitoring. In contrast, the feature attributions fed to FADMON can expose the spatial patterns driving the detected drift through attribution visualizations, at the cost of higher computational overhead. Overall, these results show that FADMON offers a favorable trade-off between detection capability and interpretability, complementing data and probability-based detectors for a comprehensive, more explainable model monitoring procedure. Figure 4 illustrates how the feature attributions FADMON uses can be visualized for better model interpretation. In drifted scenarios, the attribution maps show overall weaker activations, indicating that the model struggles more to identify strong evidence on which to base its decisions. The maps also expose the model’s limitations in multi-target scenarios: the model receives multiple strong, yet conflicting, positive and negative attributions across all objectives, suggesting that it attempts to satisfy all targets simultaneously and therefore considers multiple, potentially inconsistent, directions.

## Conclusions

In this paper we have presented FADMON, a semi-supervised concept drift detection method for visual RL. By integrating statistical tests with feature attribution techniques, FADMON enables unsupervised concept drift detection as well as supervised comprehensive model diagnosis using visual explanations. We have shown how FADMON can be integrated into the CMM cycle of DRL models such as the high-level decision-making system of SMAUG’s maritime port autonomous surveillance system. Our experiments suggest that, while incurring additional computational costs that don´t directly improve drift detection precision, FADMON can add an explainability layer to the monitoring system while also supporting detection of changes in the underlying interpretation of the input data by the DRL model, monitoring the concept rather than the data. Future work could explore the proposed methodology on other feature attribution methods or interpretable-by-design models that generate feature attributions without additional operations. Other statistical metrics could also be tested. FADMON could also be tested in other image-based DNN models such as image classifiers, detectors or generators. Finally, our method could be tested on other high-risk scenarios where continuous explainable monitoring is beneficial or even mandatory. Overall, this work shows how the combination of drift detection and XAI can enhance model monitoring in scenarios where understanding why a model fails is as important as when.

## Ethics and consent

Ethical approval and consent were not required.

## Data Availability

No source data were used for this article.

## Software availability

The synthetic data used in the experiments of this article can be accessed at the following repository:

Zenodo: Generated synthetic dataset of pixel-level representations of maritime port surveillance vessels under multiple drift scenarios

https://doi.org/10.5281/zenodo.17793696 ^47^

This project contains the following underlying data:

- background_ag1.pkl (Background of training data provided to GradientSHAP)
- reference_data_ag1.pkl (Reference input data)
- test_data_ag1.pkl (Undrifted test input data)
- data_drift_opt1_ag1.pkl (Drifted input data from the obstacle density increase scenario, variant 1)
- data_drift_opt2_ag1.pkl (Drifted input data from the obstacle density increase scenario, variant 2)
- data_drift_opt3_ag1.pkl (Drifted input data from the obstacle density increase scenario, variant 3)
- data_prob_drift_opt1_ag1.pkl (Drifted input data from the vessel confinement scenario, variant 1)
- data_prob_drift_opt2_ag1.pkl (Drifted input data from the vessel confinement scenario, variant 2)
- data_prob_drift_opt3_ag1.pkl (Drifted input data from the vessel confinement scenario, variant 3)
- ref_list_probs_ag1.pkl (Reference probability vectors)
- test_list_probs_ag1.pkl (Undrifted test probability vectors)
- list_probs_datadrift_opt1_ag1.pkl (Drifted probability vectors from the obstacle density increase scenario, variant 1)
- list_probs_datadrift_opt2_ag1.pkl (Drifted probability vectors from the obstacle density increase scenario, variant 2)
- list_probs_datadrift_opt3_ag1.pkl (Drifted probability vectors from the obstacle density increase scenario, variant 3)
- list_probs_probdrift_opt1_ag1.pkl (Drifted probability vectors from the vessel confinement scenario, variant 1)
- list_probs_probdrift_opt2_ag1.pkl (Drifted probability vectors from the vessel confinement scenario, variant 2)
- list_probs_probdrift_opt3_ag1.pkl (Drifted probability vectors from the vessel confinement scenario, variant 3)

Data is available under the terms of the Creative Commons Attribution 4.0 International license.
