# Supplementary material for: Drift detection on feature attributions for monitoring visual reinforcement learning models in maritime port surveillance
Source: Open Res Eur. 2026 Jan 2;6:2. [Version 1] doi: 10.12688/openreseurope.22116.1 (PMC12859422; doi:10.12688/openreseurope.22116.1)
Supplement: Peer review history

# Reviewer report: Reviewer response for version 1

**DOI:** 10.21956/openreseurope.23936.r67978

**Published:** 2026-01-31

**Authors:** Ahmed Mohy Ibrahim

**Affiliations:** 1 Maritime Security and Safety, Arab Academy for Science Technology and Maritime Transport, Alexandria, Alexandria Governorate, Egypt

The paper is highly promising and conceptually strong. It moves the field of 'Reliable AI' forward by treating XAI not just as a visual aid for humans but as a data stream for automated monitoring, and I suggest a few minor comments to further enhance the overall clarity and technical accuracy of the manuscript.

**General**

- Review the use of the abbreviations in the text, such as “RAPID, UNDERSEC, RL.”
- Why GradientSHAP was chosen over other methods (like LIME or Saliency Maps)

**Introduction**

- The authors should explicitly define how concept drift is established in a policy
- The introduction should acknowledge that while XAI is more expensive than raw data monitoring, the "cost of failure" in maritime port security (illegal trafficking, collisions) justifies the additional compute required for FADMON

**Related Work**

- The summary of DRL history (DQN, A3C, PPO) is long and needs to be directly related to the problem of monitoring these models (expand on the specific challenges of visual RL). How temporal dependencies (frame stacking) and high-dimensional pixel inputs make the standard drift detection method (like the KS test)

**Methodology**

- The authors should justify why spatial invariance is acceptable here. The authors state, "These values are sorted to….for feature attributions." In maritime surveillance, the location of a feature (e.g., an obstacle appearing in the vessel's direct path vs. far away) is critical to the model's logic.
- The method uses (τ=0.025τ=0.025). There is no explanation for how this value was obtained. If too high or too low. May include a sensitivity analysis (as a table or graph) showing how different values of ττ affect the detection delay or the false positive rate.
- How many samples are required to create a statistically stable "reference distribution"? If the reference set is too small, the MMD test will be subject to high variance. State the number of samples used for the reference monitoring data and provide a brief justification

**Experimental Results, and Results**

- Need justification: p-value is a probability; reporting it as exactly zero with zero variance over 30 runs?
- At what p-value or MMD distance should an operator stop the system?
- Lower mean p-values: It doesn't clearly state if FADMON outperformed them in speed of detection or just interpretability.
- Explain Figure 4: Is the model looking at the wrong things or just looking at the right things less intensely?

**Conclusion and Discussion**

- The "Plain Language Summary" mentions the "need for stronger hardware" as a warning. However, this is not mentioned in the main text. For a real-time port surveillance system, if the monitoring lag is greater than the decision-making process, the system is unsafe.

- Add a brief discussion on the "Alert Catcher" logic.

Is the study design appropriate and does the work have academic merit?

Yes

Is the work clearly and accurately presented and does it cite the current literature?

Yes

If applicable, is the statistical analysis and its interpretation appropriate?

Partly

Are all the source data underlying the results available to ensure full reproducibility?

Yes

Are the conclusions drawn adequately supported by the results?

Yes

Are sufficient details of methods and analysis provided to allow replication by others?

Partly

Reviewer Expertise:

Maritime security governance and compliance effectiveness under international regulatory frameworks, applying PhD-level research methodologies and peer-reviewed analytical approaches to develop and validate novel enhancements to conventional maritime surveillance systems for autonomous vessels, remote control centers, and smart port ecosystems, integrating AI-enabled threat modeling and immersive training to strengthen risk assessment and auditor competency evaluation

I confirm that I have read this submission and believe that I have an appropriate level of expertise to confirm that it is of an acceptable scientific standard, however I have significant reservations, as outlined above.

# Reviewer report: Reviewer response for version 1

**DOI:** 10.21956/openreseurope.23936.r67977

**Published:** 2026-01-30

**Authors:** Ayoola Babatunde Fadola

**Affiliations:** 1 Washington College of Law, American University, Washington, D.C, USA

**What works well**

1. Clear motivation: Monitoring “concepts” via how a policy interprets inputs is compelling in safety/security contexts and aligns with transparency expectations under the EU AI Act.
2. Method simplicity: A clean pipeline that’s easy to integrate into CMM.
3. Deployment-oriented architecture: The three-stage framework (reference data, online monitoring with health reports, and human-in-the-loop) is practical.
4. Qualitative insights: Attribution maps provide actionable diagnostics beyond a binary “drift/no-signal.

Key issues to address

1. Resolve data-availability inconsistency: The section states “No source data were used,” yet links to Zenodo datasets used in the experiments. Please amend to accurately reflect that all synthetic source data are available and point to the precise DOI and file mapping used for each experiment in Table 1.

Closing note

 This is a promising and timely contribution. With the statistical reporting tightened, FADMON could become a practical building block for explainable monitoring of visual RL in high-risk, safety-critical deployments.

Is the study design appropriate and does the work have academic merit?

Partly

Is the work clearly and accurately presented and does it cite the current literature?

Yes

If applicable, is the statistical analysis and its interpretation appropriate?

Partly

Are all the source data underlying the results available to ensure full reproducibility?

Partly

Are the conclusions drawn adequately supported by the results?

Yes

Are sufficient details of methods and analysis provided to allow replication by others?

Partly

Reviewer Expertise:

My research focuses on maritime law, operations, and autonomous shipping. I am able to adequately review the conversation as to the layout and operability.

I confirm that I have read this submission and believe that I have an appropriate level of expertise to confirm that it is of an acceptable scientific standard.

# Reviewer report: Reviewer response for version 1

**DOI:** 10.21956/openreseurope.23936.r67508

**Published:** 2026-01-30

**Authors:** Adeola Oluwatoyin OSUNDIRAN

**Affiliations:** 1 University of South Africa, Pretoria, Gauteng, South Africa

The research article is relevant. The problem statement was well articulated, alluding to the incessant smuggling of drugs to Europe. I would like to suggest that, inasmuch as the FADMON was highly recommended, it would have been great for the authors to highlight the challenges or applicability for other nations.

Is the study design appropriate and does the work have academic merit?

Yes

Is the work clearly and accurately presented and does it cite the current literature?

Yes

If applicable, is the statistical analysis and its interpretation appropriate?

I cannot comment. A qualified statistician is required.

Are all the source data underlying the results available to ensure full reproducibility?

Partly

Are the conclusions drawn adequately supported by the results?

Yes

Are sufficient details of methods and analysis provided to allow replication by others?

Yes

Reviewer Expertise:

Area of Expertise is on Port Efficiency and Operations. Maritime Supply Chain; Gender; Sustainability and Green Ports and Corridors

I confirm that I have read this submission and believe that I have an appropriate level of expertise to confirm that it is of an acceptable scientific standard.

# Reviewer report: Reviewer response for version 1

**DOI:** 10.21956/openreseurope.23936.r67510

**Published:** 2026-01-08

**Authors:** Alexandru Pohontu

**Affiliations:** 1 National University of Science and Technology Politehnica Bucharest, Bucharest, Romania

This paper introduces FADMON, an explainability-driven drift detector for image-based deep reinforcement learning (DRL) models, motivated by the need for continuous model monitoring (CMM) in safety/security contexts such as maritime port surveillance (SMAUG). Instead of monitoring only input images or output action probabilities, FADMON monitors feature attribution maps (e.g., GradientSHAP) to detect changes in how the model “interprets” its inputs, framed as monitoring the concept rather than only the data.

*Are sufficient details of methods and analysis provided to allow replication by others? **Partly***

 It would be helpful to specify how MMD p-values were computed. They came from a permutation test or an asymptotic approximation? What were the key settings (e.g., number of permutations, random seeds)?

 Also,the method description says the MMD value is compared to a predefined threshold to raise alerts, but the paper should specify how that threshold is chosen, whether it’s fixed across environments, and how false positive rates are controlled.

 Finally, the paper is clear, academically meaningful, provides supporting evidence for its conclusions, and shares source artifacts for the experiments.

Is the study design appropriate and does the work have academic merit?

Yes

Is the work clearly and accurately presented and does it cite the current literature?

Yes

If applicable, is the statistical analysis and its interpretation appropriate?

I cannot comment. A qualified statistician is required.

Are all the source data underlying the results available to ensure full reproducibility?

Yes

Are the conclusions drawn adequately supported by the results?

Yes

Are sufficient details of methods and analysis provided to allow replication by others?

Partly

Reviewer Expertise:

I am studying the potential of Artificial Intelligence an Machine Learning in maritime surveillance. My focus is on implementing AI in various Maritime Domain Awareness applications, including anomaly detection, risk assessment, route predictions, and collision avoidance. I review state-of-the-art methods and research novel approaches to enhance conventional maritime surveillance systems.

I confirm that I have read this submission and believe that I have an appropriate level of expertise to confirm that it is of an acceptable scientific standard.
